# 2-Phenyl­thieno[2,3-*b*]quinoxaline

**DOI:** 10.1107/S1600536812000098

**Published:** 2012-01-14

**Authors:** Youssef Ramli, Hafid Zouihri, Mohamed Azougagh, Ahmed Moussaif, El Mokhtar Essassi

**Affiliations:** a Laboratoire Nationale de Controle des Medicaments, Direction du Medicament et de la Pharmacie, BP 6206, 10000 Rabat, Morocco; b Laboratoire de Chimie Heterocyclique, Pole de Competence PHARCHIM, Université Mohammed V-Agdal, BP 1014, Rabat, Morocco; c Laboratoire de Diffraction des Rayons X, Division UATRS, Centre National pour la Recherche Scientifique et Technique, Rabat, Morocco; d Unité de la Radioimmunoanalyse, Centre National d’Etudes Scientifiques et Techniques d’Energie Nucléaire, Maamoura, Morocco

## Abstract

The title compound, C_16_H_10_N_2_S, is almost planar (r.m.s. deviation for all non-H atoms = 0.080 Å). The dihedral angle between the three fused-ring system and the phenyl ring is 9.26 (3)°. The S atom and the opposite C atom of the thio­phene ring are mutually disordered with an occupancy ratio of 0.7706 (19):0.2294 (19).

## Related literature

For a related structure, see: Ramli *et al.* (2011 ▶). For the biological activity of quinoxaline derivatives, see: Kleim *et al.* (1995 ▶). For their anti­tumour and anti­tuberculous properties, see: Abasolo *et al.* (1987 ▶); Rodrigo *et al.* (2002 ▶) and for their anti­fungal, herbicidal, anti­dyslipidemic and anti­oxidative activity, see: Jampilek *et al.* (2005 ▶); Sashidhara *et al.* (2009 ▶); Watkins *et al.* (2009 ▶).
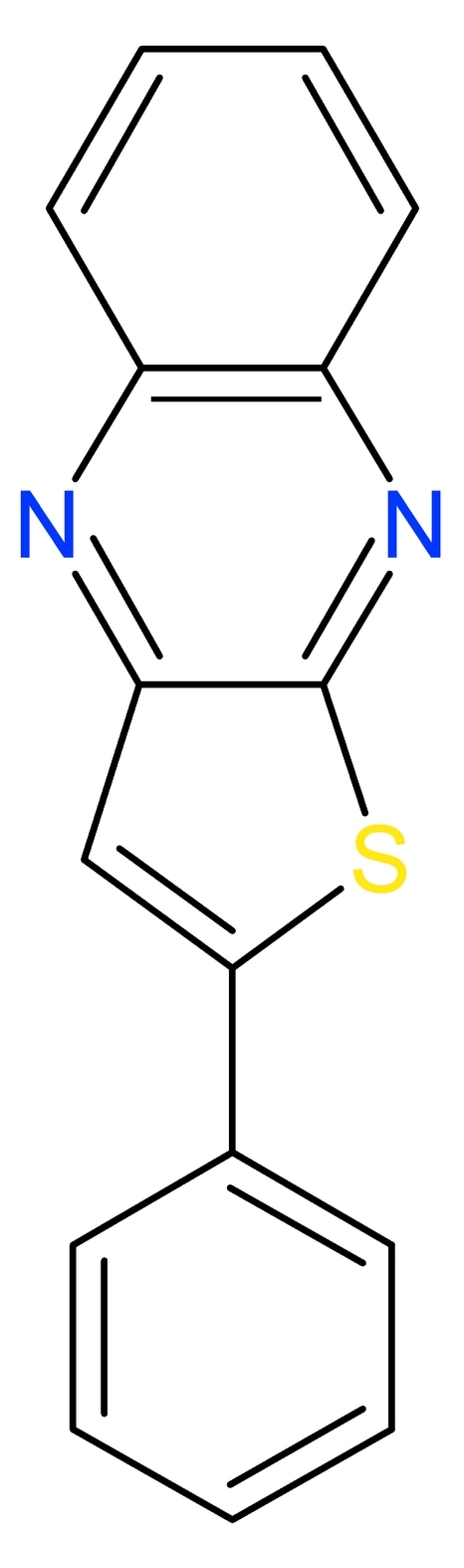


## Experimental

### 

#### Crystal data

C_16_H_10_N_2_S
*M* *_r_* = 262.32
Monoclinic, 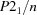

*a* = 6.3875 (3) Å
*b* = 16.2896 (8) Å
*c* = 11.6054 (6) Å
β = 96.039 (3)°
*V* = 1200.84 (10) Å^3^
*Z* = 4
Mo *K*α radiation
μ = 0.25 mm^−1^
*T* = 296 K
0.41 × 0.24 × 0.21 mm

#### Data collection

Bruker APEXII CCD detector diffractometer
27721 measured reflections
4821 independent reflections
4179 reflections with *I* > 2σ(*I*)
*R*_int_ = 0.034

#### Refinement

*R*[*F* ^2^ > 2σ(*F* ^2^)] = 0.041
*wR*(*F* ^2^) = 0.109
*S* = 1.06
4821 reflections
192 parameters
H-atom parameters constrained
Δρ_max_ = 0.58 e Å^−3^
Δρ_min_ = −0.24 e Å^−3^

### 

Data collection: *APEX2* (Bruker, 2005 ▶); cell refinement: *SAINT* (Bruker, 2005 ▶); data reduction: *SAINT*; program(s) used to solve structure: *SHELXS97* (Sheldrick, 2008 ▶); program(s) used to refine structure: *SHELXL97* (Sheldrick, 2008 ▶); molecular graphics: *PLATON* (Spek, 2009 ▶); software used to prepare material for publication: *publCIF* (Westrip, 2010 ▶).

## Supplementary Material

Crystal structure: contains datablock(s) I, global. DOI: 10.1107/S1600536812000098/bt5773sup1.cif

Structure factors: contains datablock(s) I. DOI: 10.1107/S1600536812000098/bt5773Isup2.hkl

Supplementary material file. DOI: 10.1107/S1600536812000098/bt5773Isup3.cml

Additional supplementary materials:  crystallographic information; 3D view; checkCIF report

## Figures and Tables

**Fig. 1.**
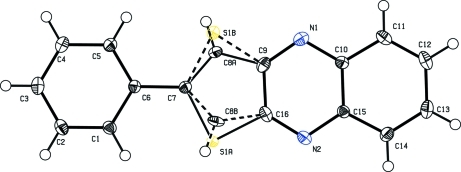
Molecular view of the title compound showing the atom-labeling scheme. Displacement ellipsoids are drawn at the 50% probability level. H atoms are represented as small spheres of arbitrary radii.

## supplementary crystallographic information

### Comment

Quinoxaline derivatives were found to exhibit antimicrobial [Kleim *et al.* 1995], antitumor [Abasolo *et al.* 1987], and antituberculous activity [Rodrigo *et al.*2002]. They, also, exhibit interesting antifungal, herbicidal, Antidyslipidemic and antioxidative activities of quinoxaline derivatives, see: [Jampilek *et al.* 2005, Sashidhara *et al.* 2009, Watkins *et al.* 2009].

In a former paper, we reported the crystal structure of 2-Methyl-3-(n-octylsulfanyl)quinoxaline [Ramli *et al.* 2011]. In this communication, the crystal structure of 2-phenyl-4a,8a-dihydrothieno[2,3-*b*]quinoxaline.

The title compound, C_16_H_10_N_2_S, shows an almost planar geometry, defined by the attached benzene [r.m.s. deviation: 0.0089 (10) A] and 4a,8a-dihydrothieno[2,3-*b*]quinoxaline groups [r.m.s. deviation: 0.2722 (9) A]. The dihedral angle between the planes of this groups is 9.26 (3)°. The S1 and C9 atoms of the thiophene ring displays 0.7706 (19): 0.2294 (19) positional disorder.

### Experimental

6.25 mmol of 3-methylquinoxaline-2-thione is merged with 12.5 mmol of the appropriate aldehyde for 2 h at the boiling point of the latter. At the end of the reaction, the solid is allowed to cool and then heated to 100° C for 10 minutes in 50 ml of ethanol. The product is filtered hot and washed with ethanol

### Refinement

The H atoms were positioned geometrically and constrained to ride on their parent atoms with C—H = 0.93Å and *U*_iso_(H) = 1.2 *U*_eq_(C).

The atoms S1 and C8 in the thiophene ring are mutually disordered by a ratio of 0.7706 (19):0.2294 (19).

### Crystal data

**Table:**

C_16_H_10_N_2_S	*F*(000) = 544
*M**_r_* = 262.32	*D*_x_ = 1.451 Mg m^−^^3^
Monoclinic, *P*2_1_/*n*	Mo *K*α radiation, λ = 0.71073 Å
Hall symbol: -P 2yn	Cell parameters from 215 reflections
*a* = 6.3875 (3) Å	θ = 1.8–26.7°
*b* = 16.2896 (8) Å	µ = 0.25 mm^−^^1^
*c* = 11.6054 (6) Å	*T* = 296 K
β = 96.039 (3)°	Prism, yellow
*V* = 1200.84 (10) Å^3^	0.41 × 0.24 × 0.21 mm
*Z* = 4	

### Data collection

**Table:**

Bruker APEXII CCD detector diffractometer	4179 reflections with *I* > 2σ(*I*)
Radiation source: fine-focus sealed tube	*R*_int_ = 0.034
graphite	θ_max_ = 34.0°, θ_min_ = 2.2°
ω and φ scans	*h* = −10→8
27721 measured reflections	*k* = −25→25
4821 independent reflections	*l* = −15→18

### Refinement

**Table:**

Refinement on *F*^2^	Primary atom site location: structure-invariant direct methods
Least-squares matrix: full	Secondary atom site location: difference Fourier map
*R*[*F*^2^ > 2σ(*F*^2^)] = 0.041	Hydrogen site location: inferred from neighbouring sites
*wR*(*F*^2^) = 0.109	H-atom parameters constrained
*S* = 1.06	*w* = 1/[σ^2^(*F*_o_^2^) + (0.0478*P*)^2^ + 0.5714*P*] where *P* = (*F*_o_^2^ + 2*F*_c_^2^)/3
4821 reflections	(Δ/σ)_max_ = 0.004
192 parameters	Δρ_max_ = 0.58 e Å^−^^3^
0 restraints	Δρ_min_ = −0.24 e Å^−^^3^

### Special details

**Table:**

Geometry. All e.s.d.'s (except the e.s.d. in the dihedral angle between two l.s. planes) are estimated using the full covariance matrix. The cell e.s.d.'s are taken into account individually in the estimation of e.s.d.'s in distances, angles and torsion angles; correlations between e.s.d.'s in cell parameters are only used when they are defined by crystal symmetry. An approximate (isotropic) treatment of cell e.s.d.'s is used for estimating e.s.d.'s involving l.s. planes.
Refinement. Refinement of *F*^2^ against ALL reflections. The weighted *R*-factor *wR* and goodness of fit *S* are based on *F*^2^, conventional *R*-factors *R* are based on *F*, with *F* set to zero for negative *F*^2^. The threshold expression of *F*^2^ > σ(*F*^2^) is used only for calculating *R*-factors(gt) *etc*. and is not relevant to the choice of reflections for refinement. *R*-factors based on *F*^2^ are statistically about twice as large as those based on *F*, and *R*- factors based on ALL data will be even larger.

### Fractional atomic coordinates and isotropic or equivalent isotropic displacement parameters (Å^2^)

**Table:**

	*x*	*y*	*z*	*U*_iso_*/*U*_eq_	Occ. (<1)
S1A	0.54001 (5)	0.93619 (2)	0.62771 (5)	0.01314 (11)	0.7706 (19)
C1	0.90893 (16)	1.05183 (6)	0.61903 (9)	0.01554 (17)	
H1	0.8109	1.0383	0.5569	0.019*	
C6	0.87615 (15)	1.02638 (6)	0.73110 (8)	0.01308 (16)	
C5	1.02452 (16)	1.04876 (6)	0.82349 (9)	0.01653 (18)	
H5	1.0041	1.0331	0.8986	0.020*	
C7	0.69078 (15)	0.97700 (6)	0.75084 (8)	0.01331 (17)	
C4	1.20213 (17)	1.09414 (6)	0.80401 (10)	0.01888 (19)	
H4	1.2993	1.1088	0.8660	0.023*	
C2	1.08669 (17)	1.09714 (6)	0.59971 (10)	0.01815 (19)	
H2	1.1066	1.1138	0.5250	0.022*	
C3	1.23453 (17)	1.11763 (6)	0.69180 (10)	0.01883 (19)	
H3	1.3547	1.1469	0.6786	0.023*	
C10	0.14188 (16)	0.83199 (6)	0.87817 (8)	0.01446 (17)	
C15	0.08412 (15)	0.82081 (6)	0.75707 (8)	0.01349 (16)	
C9	0.42799 (16)	0.90599 (6)	0.83950 (10)	0.01732 (18)	
C14	−0.10173 (16)	0.77645 (6)	0.71932 (9)	0.01765 (19)	
H14	−0.1401	0.7688	0.6405	0.021*	
C16	0.36948 (16)	0.89267 (6)	0.71805 (9)	0.01666 (18)	
C11	0.01306 (17)	0.79725 (7)	0.95760 (9)	0.01880 (19)	
H11	0.0504	0.8032	1.0368	0.023*	
C12	−0.16630 (18)	0.75488 (7)	0.91879 (11)	0.0211 (2)	
H12	−0.2500	0.7326	0.9718	0.025*	
C13	−0.22485 (17)	0.74484 (6)	0.79834 (11)	0.0205 (2)	
H13	−0.3476	0.7166	0.7728	0.025*	
N2	0.20272 (14)	0.85147 (5)	0.67565 (8)	0.01643 (16)	
N1	0.31701 (15)	0.87565 (6)	0.91971 (8)	0.01824 (17)	
C8A	0.6155 (2)	0.95601 (9)	0.8506 (2)	0.0156 (3)	0.7706 (19)
H8A	0.6801	0.9727	0.9223	0.019*	0.7706 (19)
S1B	0.6418 (2)	0.96348 (8)	0.89788 (17)	0.0172 (4)	0.2294 (19)
C8B	0.5408 (8)	0.9415 (3)	0.6794 (7)	0.0181 (9)	0.2294 (19)
H8B	0.5454	0.9481	0.6002	0.022*	0.2294 (19)

### Atomic displacement parameters (Å^2^)

**Table:**

	*U*^11^	*U*^22^	*U*^33^	*U*^12^	*U*^13^	*U*^23^
S1A	0.01306 (14)	0.01609 (14)	0.0103 (2)	−0.00218 (10)	0.00130 (12)	0.00077 (12)
C1	0.0167 (4)	0.0156 (4)	0.0146 (4)	0.0001 (3)	0.0033 (3)	−0.0008 (3)
C6	0.0123 (4)	0.0128 (3)	0.0144 (4)	0.0005 (3)	0.0020 (3)	−0.0005 (3)
C5	0.0156 (4)	0.0175 (4)	0.0162 (4)	−0.0015 (3)	0.0000 (3)	0.0002 (3)
C7	0.0127 (4)	0.0139 (3)	0.0132 (4)	0.0002 (3)	0.0005 (3)	0.0000 (3)
C4	0.0154 (4)	0.0179 (4)	0.0229 (5)	−0.0021 (3)	0.0001 (4)	−0.0012 (3)
C2	0.0199 (4)	0.0162 (4)	0.0197 (5)	−0.0003 (3)	0.0081 (4)	0.0001 (3)
C3	0.0159 (4)	0.0146 (4)	0.0268 (5)	−0.0009 (3)	0.0062 (4)	−0.0016 (3)
C10	0.0148 (4)	0.0150 (4)	0.0137 (4)	0.0015 (3)	0.0019 (3)	0.0004 (3)
C15	0.0137 (4)	0.0134 (3)	0.0134 (4)	0.0003 (3)	0.0016 (3)	0.0015 (3)
C9	0.0133 (4)	0.0158 (4)	0.0221 (5)	0.0005 (3)	−0.0012 (3)	−0.0012 (3)
C14	0.0165 (4)	0.0164 (4)	0.0193 (5)	−0.0014 (3)	−0.0018 (3)	0.0013 (3)
C16	0.0149 (4)	0.0162 (4)	0.0197 (5)	0.0023 (3)	0.0057 (3)	0.0051 (3)
C11	0.0207 (5)	0.0209 (4)	0.0157 (4)	0.0037 (4)	0.0059 (4)	0.0033 (3)
C12	0.0190 (5)	0.0192 (4)	0.0268 (5)	0.0024 (4)	0.0103 (4)	0.0071 (4)
C13	0.0151 (4)	0.0161 (4)	0.0300 (6)	−0.0019 (3)	0.0012 (4)	0.0035 (4)
N2	0.0175 (4)	0.0173 (4)	0.0149 (4)	0.0016 (3)	0.0037 (3)	0.0027 (3)
N1	0.0170 (4)	0.0197 (4)	0.0175 (4)	0.0008 (3)	−0.0009 (3)	−0.0023 (3)
C8A	0.0148 (6)	0.0193 (6)	0.0120 (7)	−0.0013 (4)	−0.0015 (5)	−0.0012 (5)
S1B	0.0150 (5)	0.0205 (5)	0.0160 (8)	−0.0038 (4)	0.0006 (5)	0.0002 (5)
C8B	0.022 (2)	0.023 (2)	0.008 (2)	0.0030 (16)	0.0028 (19)	−0.0009 (18)

### Geometric parameters (Å, °)

**Table:**

S1A—C16	1.7403 (11)	C10—C15	1.4267 (14)
S1A—C7	1.7669 (10)	C15—N2	1.3663 (13)
C1—C2	1.3921 (15)	C15—C14	1.4192 (14)
C1—C6	1.4017 (14)	C9—N1	1.3236 (15)
C1—H1	0.9300	C9—C16	1.4362 (15)
C6—C5	1.4024 (14)	C9—C8A	1.4430 (19)
C6—C7	1.4692 (14)	C9—S1B	1.7338 (18)
C5—C4	1.3923 (15)	C14—C13	1.3698 (16)
C5—H5	0.9300	C14—H14	0.9300
C7—C8B	1.331 (7)	C16—N2	1.3103 (14)
C7—C8A	1.344 (2)	C16—C8B	1.460 (6)
C7—S1B	1.781 (2)	C11—C12	1.3722 (16)
C4—C3	1.3935 (16)	C11—H11	0.9300
C4—H4	0.9300	C12—C13	1.4179 (17)
C2—C3	1.3903 (16)	C12—H12	0.9300
C2—H2	0.9300	C13—H13	0.9300
C3—H3	0.9300	C8A—H8A	0.9300
C10—N1	1.3697 (13)	C8B—H8B	0.9300
C10—C11	1.4165 (14)		
			
C16—S1A—C7	89.34 (5)	C14—C15—C10	119.36 (9)
C2—C1—C6	120.68 (9)	N1—C9—C16	122.04 (9)
C2—C1—H1	119.7	N1—C9—C8A	130.45 (13)
C6—C1—H1	119.7	C16—C9—C8A	107.50 (12)
C1—C6—C5	118.53 (9)	N1—C9—S1B	112.64 (10)
C1—C6—C7	120.54 (9)	C16—C9—S1B	125.31 (10)
C5—C6—C7	120.93 (9)	C8A—C9—S1B	17.82 (8)
C4—C5—C6	120.70 (10)	C13—C14—C15	120.34 (10)
C4—C5—H5	119.7	C13—C14—H14	119.8
C6—C5—H5	119.7	C15—C14—H14	119.8
C8B—C7—C8A	97.3 (3)	N2—C16—C9	124.28 (9)
C8B—C7—C6	132.8 (3)	N2—C16—C8B	140.3 (3)
C8A—C7—C6	129.87 (12)	C9—C16—C8B	95.3 (3)
C8B—C7—S1A	15.8 (3)	N2—C16—S1A	121.20 (8)
C8A—C7—S1A	112.77 (10)	C9—C16—S1A	114.52 (8)
C6—C7—S1A	117.35 (7)	C8B—C16—S1A	19.4 (3)
C8B—C7—S1B	110.8 (3)	C12—C11—C10	120.63 (10)
C8A—C7—S1B	13.58 (8)	C12—C11—H11	119.7
C6—C7—S1B	116.29 (8)	C10—C11—H11	119.7
S1A—C7—S1B	126.35 (7)	C11—C12—C13	120.40 (10)
C5—C4—C3	120.08 (10)	C11—C12—H12	119.8
C5—C4—H4	120.0	C13—C12—H12	119.8
C3—C4—H4	120.0	C14—C13—C12	120.42 (10)
C3—C2—C1	120.20 (10)	C14—C13—H13	119.8
C3—C2—H2	119.9	C12—C13—H13	119.8
C1—C2—H2	119.9	C16—N2—C15	114.55 (9)
C2—C3—C4	119.79 (10)	C9—N1—C10	115.11 (9)
C2—C3—H3	120.1	C7—C8A—C9	115.82 (17)
C4—C3—H3	120.1	C7—C8A—H8A	122.1
N1—C10—C11	119.16 (9)	C9—C8A—H8A	122.1
N1—C10—C15	122.00 (9)	C9—S1B—C7	84.43 (10)
C11—C10—C15	118.84 (9)	C7—C8B—C16	124.0 (6)
N2—C15—C14	118.63 (9)	C7—C8B—H8B	118.0
N2—C15—C10	122.00 (9)	C16—C8B—H8B	118.0

## References

[bb1] Abasolo, M. I., Gaozza, C. H. & Fernandez, B. M. (1987). *J. Heterocycl. Chem* **24**, 1771–1775.

[bb2] Bruker (2005). *APEX2* and *SAINT* Bruker AXS Inc., Madison, Wisconsin, USA.

[bb3] Jampilek, J., Dolezal, M., Kunes, J., Buchta, V. & Kralova, K. (2005). *Med. Chem.* **1**, 591–599. 10.2174/157340605774598108 16787342

[bb4] Kleim, J. P., Bender, R., Kirsch, R., Meichsner, C., Paessens, A., Rosner, M., Rubsamen Waigmann, H., Kaiser, R., Wichers, M., Schneweis, K. E., Winkler, I. & Riess, G. (1995). *Antimicrob. Agents Chemother.* **39**, 2253–2257. 10.1128/aac.39.10.2253 PMC162925 8619578

[bb5] Ramli, Y., Moussaif, A., Zouihri, H., Bourichi, H. & Essassi, E. M. (2011). *Acta Cryst.* E**67**, o1374. 10.1107/S160053681101703X PMC3120384 21754764

[bb6] Rodrigo, G. A., Robinshon, A. E., Hedrera, M. E., Kogan, M., Sicardi, S. M. & Fernaandez, B. M. (2002). *Trends Heterocycl. Chem.* **8**, 137–143.

[bb7] Sashidhara, K. V., Kumar, A., Bhatia, G., Khan, M. M., Khanna, A. K. & Saxena, J. K. (2009). *Eur. J. Med. Chem.* **44**, 1813–1818. 10.1016/j.ejmech.2008.08.004 18819730

[bb8] Sheldrick, G. M. (2008). *Acta Cryst.* A**64**, 112–122. 10.1107/S0108767307043930 18156677

[bb9] Spek, A. L. (2009). *Acta Cryst.* D**65**, 148–155. 10.1107/S090744490804362X PMC2631630 19171970

[bb10] Watkins, A. J., Nicol, G. W. & Shawa, L. J. (2009). *Soil Biol. Biochem.* **41**, 580–585.

[bb11] Westrip, S. P. (2010). *J. Appl. Cryst.* **43**, 920–925.

